# Taxonomic reassessment of two subspecies of Chinese skink in Taiwan based on morphological and molecular investigations (Squamata, Scincidae)

**DOI:** 10.3897/zookeys.687.12742

**Published:** 2017-08-02

**Authors:** Kazuki Kurita, Yukiko Nakamura, Taku Okamoto, Si-Min Lin, Tsutomu Hikida

**Affiliations:** 1 Department of Zoology, Graduate School of Science, Kyoto University, Kitashirakawa-oiwakecho, Sakyo-ku, Kyoto 606-8502, Japan; 2 Department of Animal Development and Physiology, Graduate School of Biostudies, Kyoto University, Yoshida-Konoecho, Sakyo-ku, Kyoto 606-8501, Japan; 3 Department of Life Science, National Taiwan Normal University, No. 88, Sec. 4, Tingzhou Road, Taipei 111, Taiwan

**Keywords:** Green Island, *Plestiodon chinensis*, *Plestiodon leucostictus*, subspecies, Taiwan, taxonomy

## Abstract

The Chinese skink, *Plestiodon chinensis* (Gray, 1838), is widely distributed across continental China, Taiwan, the Korean Peninsula, and offshore islets, and consists of several subspecies. Here morphological and molecular methods have been used to reassess the taxonomic status and distributions of *P. c. formosensis* (Van Denburgh, 1912) and *P. c. leucostictus* (Hikida, 1988), which are endemic to Taiwan and Green Island (an islet off the east coast of Taiwan), respectively. It can be confirmed that the eastern Taiwanese populations of *P. c. formosensis* exhibit similar juvenile color patterning and genetic composition to the islet subspecies *P. c. leucostictus*, and are distinct from consubspecific populations in western Taiwan. Therefore, the eastern Taiwanese populations are assigned to *P. c. leucostictus*, and this subspecies is recognized as a distinct species, *Plestiodon leucostictus* (Hikida, 1988), based on their unique juvenile coloration and highly divergent DNA sequences. Our results also revealed that *P. c. formosensis* in western Taiwan is close to nominotypical subspecies from the continent, suggesting the necessity of a comprehensive taxonomic analysis in the future.

## Introduction

The scincid lizard genus *Plestiodon* Duméril & Bibron, 1839, comprises some 45 species, is distributed across East Asia and North America (Brandley et al. 2012, Feria-Ortiz and García-Vázquez 2012, Okamoto and Hikida 2012, Kurita and Hikida 2014a, b). Among these, *P. chinensis* (Gray, 1838) is a large-sized skink widely distributed in Asia including continental China, Taiwan, the Korean Peninsula, and islands offshore from these areas (Hikida 1993). Several available names have been given to the different geographic entities of *P. chinensis*, but their taxonomic status remain uncertain due to a paucity of studies on geographic variation in the complex. At least three subspecies have been commonly recognized by recent researchers: *P. c. chinensis* (Gray, 1838), *P. c. formosensis* (Van Denburgh, 1912), and *P. c. leucostictus* (Hikida, 1988) (Hikida 1993, Zhao and Adler 1993, Zhao et al. 1999), but the recognition of two additional subspecies, *P. c. pulcher* (Duméril & Bibron, 1839) and *P. c. daishanensis* (Mao, 1983), varies among different authors.

Two subspecies, *P. c. formosensis* and *P. c. leucostictus*, are known from Taiwan and its adjacent islets. Van Denburgh (1912a,b) first described the former subspecies as *Eumeces chinensis formosensis* from “San Shi Ka, Formosa” (see Discussion for current name and location), on the basis of differences in head scutellation and body coloration from the nominotypical subspecies. In a revision of *Eumeces* *sensu lato* (encompassing *Plestiodon* of the current classification), Taylor (1936) considered this subspecies a junior synonym of *P. c. chinensis*. However, subsequent researchers (e.g. Hikida 1993, Zhao and Adler 1993, Zhao et al. 1999) recognized its validity. It has been known to occur in lowland areas (less than 500 m) of Taiwan, with a large disjunction across the central mountain ranges which separates the island into the eastern and western geographic zones (Shang et al. 2009). The second subspecies was described by Hikida (1988) as *Eumeces chinensis leucostictus* from Green Island (also known as Ludao, Lyudao, or Lutao), located off the southeast of Taiwan. It is clearly distinct from the other subspecies of *P. chinensis* in having a white-spotted dorsal pattern in juveniles, in addition to some scutellation differences such as a higher number of scale rows around midbody, and the usual presence of postnasal (Hikida 1988). Currently it is known to occur only on this tiny islet (15.1 km^2^), and is regarded as one of the most threatened taxon in *Plestiodon* because of the extremely restricted distribution.

However, *P. c. leucostictus*-like forms have been reported outside of Green Island. Pictures in a field report from Yu (1994) and a field guide by Shang et al. (2009), both taken in Hualien County of eastern Taiwan, indicated that *Plestiodon* individuals in this region have a white-spotted pattern on the dorsum. The geographic proximity of this area to Green Island, in conjunction with the similarity in juvenile color patterning, implies that eastern Taiwanese populations currently assigned to *P. c. formosensis* might be geographic variants of *P. c. leucostictus*. However, this debate remains because of the scarcity of specimens from eastern Taiwan. Here we reassess the taxonomic status and distributions of *P. c. formosensis* and *P. c. leucostictus* by comparing the morphological features and DNA sequences of *P. chinensis* from representative localities in Taiwan and Green Island.

## Materials and methods

### Study site and material examined

**Figure 1.**
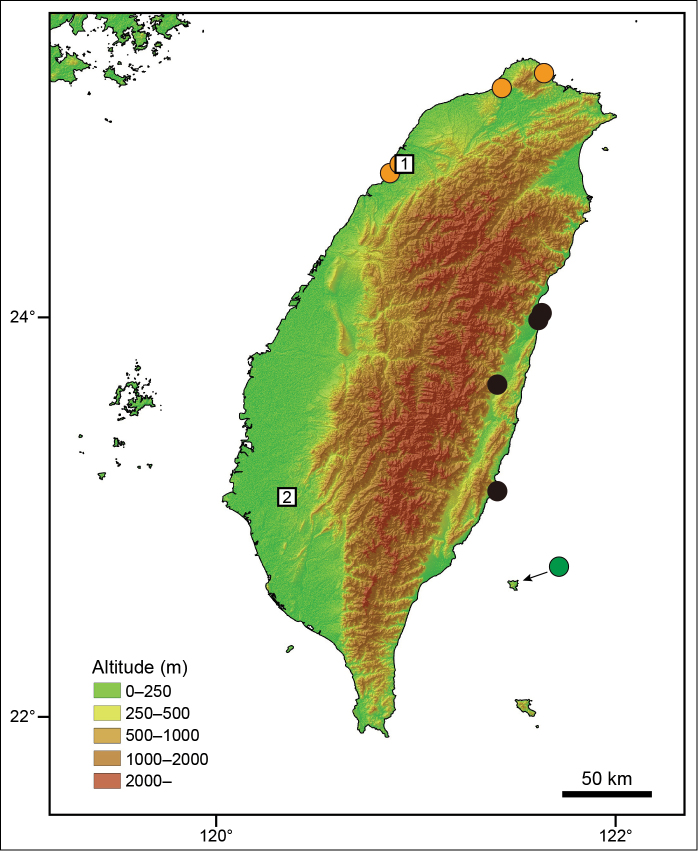
Locality map of *Plestiodon chinensis* samples used for molecular DNA genetic analysis. Orange circles: *P. c. formosensis* from western Taiwan. Black circles: *P. c.* “*formosensis*” from eastern Taiwan. Green circle: *P. c. leucostictus* from Green Island. These colors correspond to those of Figures 3 and 4. Open squares indicate the putative type locality for *P. c. formosensis* (**1** Xiangshan in Hsinchu County [Shang et al. 2009] **2** Shanshang in Tainan County [Zhao and Adler 1993]).

Specimens of *P. chinensis* from each of three regions (western Taiwan, eastern Taiwan, and Green Island) were assigned to a single operational taxonomic unit (OTU), and their taxonomic relationship was examined using morphological and molecular data. For the morphological examination, 69 specimens from Taiwan were used, including the type series of *P. c. formosensis*; and 54 specimens of *P. c. leucostictus*, including 27 newly investigated specimens and published data on the type series (Hikida 1988) (Suppl. material 1: Appendix I). In the molecular genetic analysis, representatives of each OTU were used, including specimens from the putative type locality of *P. c. formosensis* (Table 1; Fig. 1).

### Morphological examination

Three morphological features reported to distinguish *P. c. leucostictus* from other subspecies of *P. chinensis* (Hikida 1988) were characterized. These features included juvenile color pattern on dorsal and lateral regions of body (JCP), the number of scale rows around midbody (MSR), and presence or absence of postnasal scale (PN). To investigate body coloration, preserved specimens were examined. Other scutellation characters were counted and observed using a stereomicroscope where necessary.

### Molecular genetic analysis

DNA sequences of three independently evolving regions were determined for representative specimens of *P. chinensis* from Taiwan and Green Island. Two fragments of mitochondrial DNA (mtDNA) loci were sequenced for all materials. The first mtDNA fragment (termed mtDNA-1) included the 3′ end of the transfer RNA^Glu^ gene (tRNA^Glu^) and a portion of cytochrome *b* gene (cyt *b*). The second mtDNA fragment (termed mtDNA-2) included a portion of the 3′ end of the 16S ribosomal RNA gene, tRNA^Leu^, the first unit of the NADH dehydrogenase gene (ND1), tRNA^Ile^, tRNA^Gln^, and a portion of the 5′ end of tRNA^Met^. Two nuclear loci, the recombination activating gene-1 (RAG-1) and prolactin receptor (PRLR), were also sequenced for some specimens. These genes were chosen based on several relevant taxonomic and phylogenetic studies of *Plestiodon* (e.g. Brandley et al. 2012; Kurita and Toda 2017).

Total genomic DNA was extracted from liver or tail tissues stored at −80 °C or in 99% ethanol. Extractions were performed with the DNeasy Blood & Tissue kit (QIAGEN, Hilden, Germany) or by using a slightly modified version of Okamoto et al.’s (2006) method. Polymerase chain reactions (PCR) were conducted using the TaKaRa Ex Taq kit (Takara Bio, Otsu, Japan) with a GeneAmp PCR Systems 2700 machine (Applied Biosystems, Foster City, CA, USA). The PCR cycle condition for the mtDNA-1 was as follows: initial denaturation at 95°C for 5 min, followed by 30 cycles of 94°C for 1 min, 45 or 50°C for 1 min, and 70°C for 2 min, with a final extension at 72°C for 7 min. The PCR cycle condition for the mtDNA-2 was as follows: initial denaturation at 94°C for 2 min, followed by 30 cycles of 94°C for 30 s, 55°C for 30 s, and 72°C for 1 min 30 s, with a final extension at 72°C for 5 min. Amplification protocols for nuclear RAG-1 and PRLR were the same as those used by Kurita and Hikida (2014b) and Townsend et al. (2008), respectively. The amplified DNA fragments were purified by polyethylene glycol (PEG) precipitation using 0.6 volume of PEG solution (20% PEG 6000, 2.5 M NaCl). Sequencing reactions were performed using the BigDye Terminator v3.1 Cycle Sequencing Kit (Applied Biosystems). The products were cleaned by ethanol precipitation and sequenced on an Applied Biosystems 3130*xl* Genetic Analyzer (Applied Biosystems). The primers used for PCR and for sequencing are listed in Suppl. material 1: Appendix II. We used FinchTV 1.4.0 (Geospiza, http://www.geospiza.com/ Products/finchtv.shtml) and MEGA5.2.1 (Tamura et al. 2011) to view and align the sequences. Newly obtained sequence data were deposited in GenBank (accession nos. LC200983–LC201042; Table 1).

**Table 1.** Information for samples of *Plestiodon chinensis* and its relatives examined in DNA analyses. Museum abbreviations follow Sabaj Perez (2014), with the exception of MCB (Matthew C. Brandley, private collection). Accession numbers with an asterisk show newly obtained sequences in this study.

Taxon	Locality	Voucher	GenBank accession number				Source
			Cyt *b*	ND1	RAG–1	PRLR	
*P. c. formosensis*	Jinshan, New Taipei City, Taiwan	KUZ R71772	LC147548	LC147646	-	-	Kurita and Toda (2017)
*P. c. formosensis*	Jinshan, New Taipei City, Taiwan	KUZ R71780	LC200983*	LC201001*	LC201019*	LC201031*	This study
*P. c. formosensis*	Jinshan, New Taipei City, Taiwan	KUZ R71794	LC200984*	LC201002*	LC201020*	LC201032*	This study
*P. c. formosensis*	Jinshan, New Taipei City, Taiwan	KUZ R71943	LC200985*	LC201003*	-	-	This study
*P. c. formosensis*	Bali, New Taipei City, Taiwan	KUZ R69425	LC200986*	LC201004*	LC201021*	LC201033*	This study
*P. c. formosensis*	Xiangshan, Hsinchu County, Taiwan	KUZ R69417	LC200987*	LC201005*	LC201022*	LC201034*	This study
*P. c. formosensis*	Xiangshan, Hsinchu County, Taiwan	KUZ R69418	LC200988*	LC201006*	-	-	This study
*P. c. formosensis*	Xiangshan, Hsinchu County, Taiwan	KUZ R69419	LC200989*	LC201007*	-	-	This study
*P. c. formosensis*	Qiding, Miaoli County, Taiwan^†^	MCB 675	-	HM160800	HM161178	HM160896	Brandley et al. (2012)
*P. c.* “*formosensis*”	Hualien City, Hualien County, Taiwan	KUZ R60584	LC200990*	LC201008*	-	-	This study
*P. c.* “*formosensis*”	Hualien City, Hualien County, Taiwan	KUZ R69420	LC200991*	LC201009*	LC201023*	LC201035*	This study
*P. c.* “*formosensis*”	Hualien City, Hualien County, Taiwan	KUZ R69421	LC200992*	LC201010*	LC201024*	LC201036*	This study
*P. c.* “*formosensis*”	Hualien City, Hualien County, Taiwan	KUZ R69422	LC200993*	LC201011*	-	-	This study
*P. c.* “*formosensis*”	Guangfu, Hualien County, Taiwan	KUZ R69423	LC200994*	LC201012*	LC201025*	LC201037*	This study
*P. c.* “*formosensis*”	Guangfu, Hualien County, Taiwan	KUZ R69424	LC200995*	LC201013*	LC201026*	LC201038*	This study
*P. c.* “*formosensis*”	Sansiantai, Taitung County, Taiwan	KUZ R71777	LC147549	LC147647	LC201027*	LC201039*	Kurita and Toda (2017) /This study
*P. c.* “*formosensis*”	Sansiantai, Taitung County, Taiwan	KUZ R71797	LC200996*	LC201014*	-	-	This study
*P. c.* “*formosensis*”	Sansiantai, Taitung County, Taiwan	KUZ R71819	LC200997*	LC201015*	-	-	This study
*P. c.* “*formosensis*”	Sansiantai, Taitung County, Taiwan	KUZ R71822	LC200998*	LC201016*	LC201028*	LC201040*	This study
*P. c. leucostictus*	Green Island, Taitung County, Taiwan	KUZ R60571	LC200999*	LC201017*	LC201029*	LC201041*	This study
*P. c. leucostictus*	Green Island, Taitung County, Taiwan	KUZ R60581	LC201000*	LC201018*	LC201030*	LC201042*	This study
*P. c. chinensis*	Lishui, Zhejiang Province, China	-	KT279358	KT279358	-	-	Zhang et al. (2016)
*P. c. chinensis*	Nan Ao Island, Guangdong Province, China	MCZ Z39481	-	HM160801	HM161179	HM160897	Brandley et al. (2012)
*P. kishinouyei*	Miyako Island, Okinawa Prefecture, Japan	Miy120318	LC147467	LC147565	-	-	Kurita and Toda (2017)
*P. kishinouyei*	Ishigaki Island, Okinawa Prefecture, Japan	MCB 658	-	-	HM161200	HM160918	Brandley et al. (2012)
*P. tamdaoensis*	Unknown locality (pet-traded)	KUZ R66879	LC147554	LC147652	-	-	Kurita and Toda (2017)
*P. quadrilineatus*	Cheung Chau Island, Hong Kong, China	KUZ R36541	LC147555	LC147653	-	-	Kurita and Toda (2017)

^†^The locality has been listed as “Hsingchu Province: road near Ji-Ding train station: 24.72158, 120.87086” in Brandley et al. (2012), but the GPS coordinate in that paper did not correspond with this locality.

Sequences from previous studies of *P. chinensis* and its relatives were included in the analyses, including two other members of the *P. chinensis* species group (*P. kishinouyei* [Stejneger] and *P. tamdaoensis* [Bourret]), with a more distantly related species *P. quadrilineatus* Blyth used as outgroup for phylogenetic inferences (Brandley et al. 2012, Zhang et al. 2016, Kurita and Toda 2017) (Table 1). The nuclear DNA sequence dataset included *P. kishinouyei*, which was regarded as the most closely related clade of the ingroup taxa (see Results).

Mitochondrial DNA genealogy was inferred using maximum likelihood (ML) and Bayesian inference (BI) methods with TREEFINDER (March 2011 version; Jobb 2011) and MrBayes 3.2.2 (Ronquist et al. 2012), respectively. Each gene was aligned separately using MUSCLE (Edgar 2004) implemented in MEGA5 with default parameters. We then manually adjusted for rRNA and tRNA gene sequences based on their secondary structures, utilizing information from Gutell and Fox (1988) and Kumazawa and Nishida (1993), respectively. Neither stop codons nor insertions/deletions (indels) were found in the cyt *b* and ND1 gene sequences. Regions with gaps or ambiguous alignment were excluded from rRNA and tRNA sequence alignments. Dataset (total sequence of 2,351 bp) was partitioned according to genes (four tRNA regions were combined into a single partition) and codon positions (for cyt *b* and ND1). The appropriate substitution models for these datasets were selected with KAKUSAN4 (Tanabe 2011) under the corrected Akaike Information Criterion (AICc; Sugiura 1978) (Suppl. material 1: Appendix III). Statistic support for the inferred ML tree topology was assessed by using nonparametric bootstrap analysis (Felsenstein 1985) with 1,000 pseudoreplicates. In the BI analysis, we performed two independent runs of four Markov chains for 4 × 10^6^ generations per run, sampling a tree every 100 generations. After checking the adequacy (the effective sample size ≥200) of the parameter estimates and convergence using Tracer 1.5 (Rambaut and Drummond 2007), we discarded the first 20,001 trees as burin-in and calculated a consensus topology and Bayesian posterior probabilities for the remaining 40,000 trees. Statistic supports with bootstrap values of ≥70%, or Bayesian posterior probabilities of ≥95%, were regarded as sufficiently resolved for ML analysis (Hillis and Bull 1993) and BI analysis (Leaché and Reeder 2002, Huelsenbeck and Rannala 2004), respectively.

Prior to the nuclear DNA analysis, gametic phases for the individuals that were heterozygous at more than one nucleotide position were inferred using PHASE 2.1.1 (Stephens et al. 2001, Stephens and Scheet 2005). We used SeqPHASE (Flot 2010) to interconvert PHASE input/output files and performed the PHASE analysis using default settings, with the exception of setting the probability threshold to 0.7 (Harrigan et al. 2008). Five independent runs were performed with different seeds to check the consistency of the haplotype frequency estimates and pseudo-likelihood scores across runs. The absence of indels or stop codons for inferred sequences was checked by translating them with the universal nuclear genetic code using MEGA5 (Tamura et al. 2011).

We inferred gene genealogy among the detected alleles of the two nuclear loci using median-joining analyses with NETWORK 5.0.0.0 (http://www.fluxus-engineering.com; Bandelt et al. 1999). In addition, we used the nuclear DNA dataset to perform an analysis of molecular variance (AMOVA; Excoffier et al. 1992) using Arlequin 3.5.1.2 (Excoffier and Lischer 2010) with 10,000 permutations to compare the current (western and eastern Taiwan vs. Green Island) and revised (western Taiwan vs. eastern Taiwan + Green Island; see Results) classifications.

## Results

### Morphological analyses

**Figure 2.**
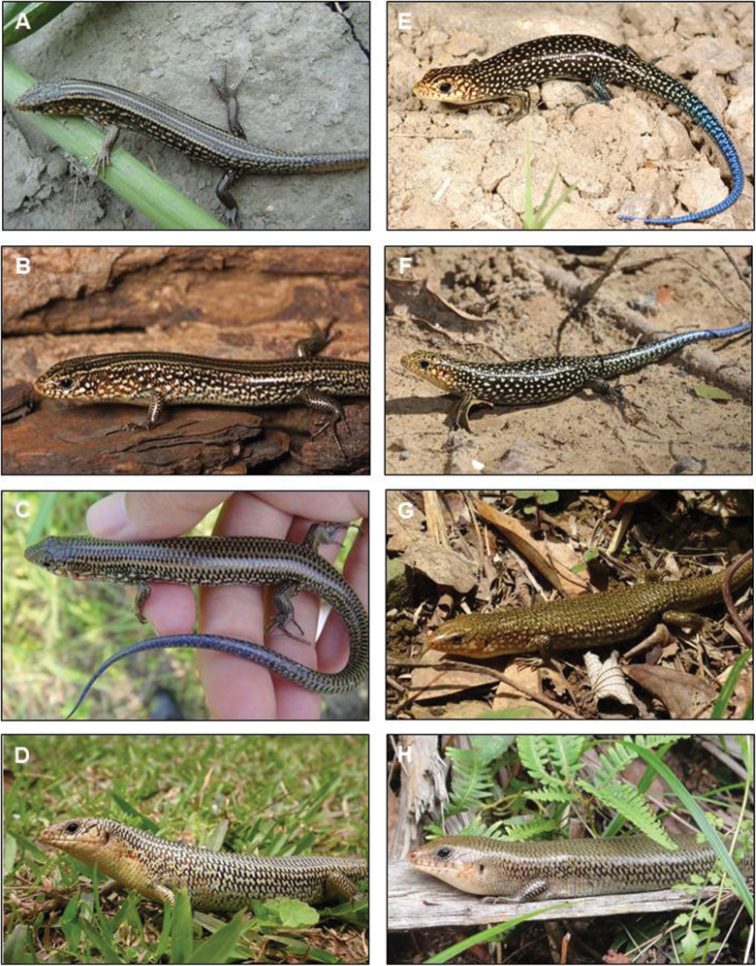
Color pattern alteration of *Plestiodon chinensis formosensis* (left, from western Taiwan) and *P. c. leucostictus* (right, from Green Island). Hatchlings (**A** vs. **E**), juveniles (**B** and **C** v.s. **F** and **G**), and adults (**D** v.s. **H**). Photographed by H.-Y. Tseng **A, C, G, H**; R.-J. Wang **B, F**; S.-M. Lin **D**; and C.-W. You **E**.

An examination of color variation demonstrated that specimens from the different focal areas had different color patterns. Almost all juvenile specimens (SVL = 47.9–60.4 mm; n = 9) from eastern Taiwan (Taitung County) showed a pattern of small white spots without light lines on the dorsum (Fig. 2). This is very similar to the color pattern of *P. c. leucostictus* described by Hikida (1988). Among these specimens, the white spotted pattern in larger juveniles was faded on the dorsal regions. One exceptional specimen (SVL = 57.6 mm) possessed white spots together with an obscure middorsal light line on the dorsum. In contrast, juveniles (SVL = 40.1–69.9 mm; n = 7) from northwestern Taiwan (New Taipei City and Miaoli County) clearly had three light lines on the dorsum and white spots on the flank (Fig. 2). This pattern was retained in the type specimens of *P. c. formosensis*, although they are adults.

Eastern Taiwanese specimens usually had 24 scale rows at midbody (range = 24–26, mean ± SD = 24.3 ± 0.7, n = 25) (Table 2). The range and mode of MSR were similar to those of western Taiwanese specimens (23–26, 24.3 ± 0.8, n = 38), while the mode was lower than that of Green Island specimens (24–27, 25.8 ± 0.7, n = 54).

Most of the eastern Taiwanese specimens examined possessed a postnasal on both sides (92%; Table 2). The frequency of the presence on both sides was higher than that of Green Island specimens (59%) and that of the western Taiwanese specimens (28%). The western Taiwanese specimens usually lack a postnasal on both sides (60%).

**Table 2.** Variation in scutellation characteristics (MSR, the number of scale rows around midbody; PN, the presence of a postnasal: A = absent or P = present) of *Plestiodon chinensis* in Taiwan and Green Island.

Locality	MSR						PN (left-right)				
	N	23	24	25	26	27	N	A-A	A-P	P-A	P-P
*P. c. formosensis*, western Taiwan											
Keelung County	3		2^P^		1		3	3^P^			
New Taipei City	17	1	15		1		17	9		4	4
Taipei City	6		4		2		6	6			
Hsinchu County	5		4^P^		1^H^		6	3^P^		1	2^HP^
Miaoli County	10		8		2		11	5			6
Total	41	1	33		7		43	26		5	12
*P. c. formosensis*, eastern Taiwan											
Hualien County	4		3		1		5		1		4
Taitung County	21		18	1	2		21			1	20
Total	25		21	1	3		26		1	1	24
*P. c. leucostictus*											
Green Island^†^	54		6	2	45	1	54	16	1	5	32

^H^Including holotype of *P. c. formosensis*; ^P^Including paratype of *P. c. formosensis* ^†^Data including the type series from Hikida (1988)

### Molecular phylogenetic analyses

**Figure 3.**
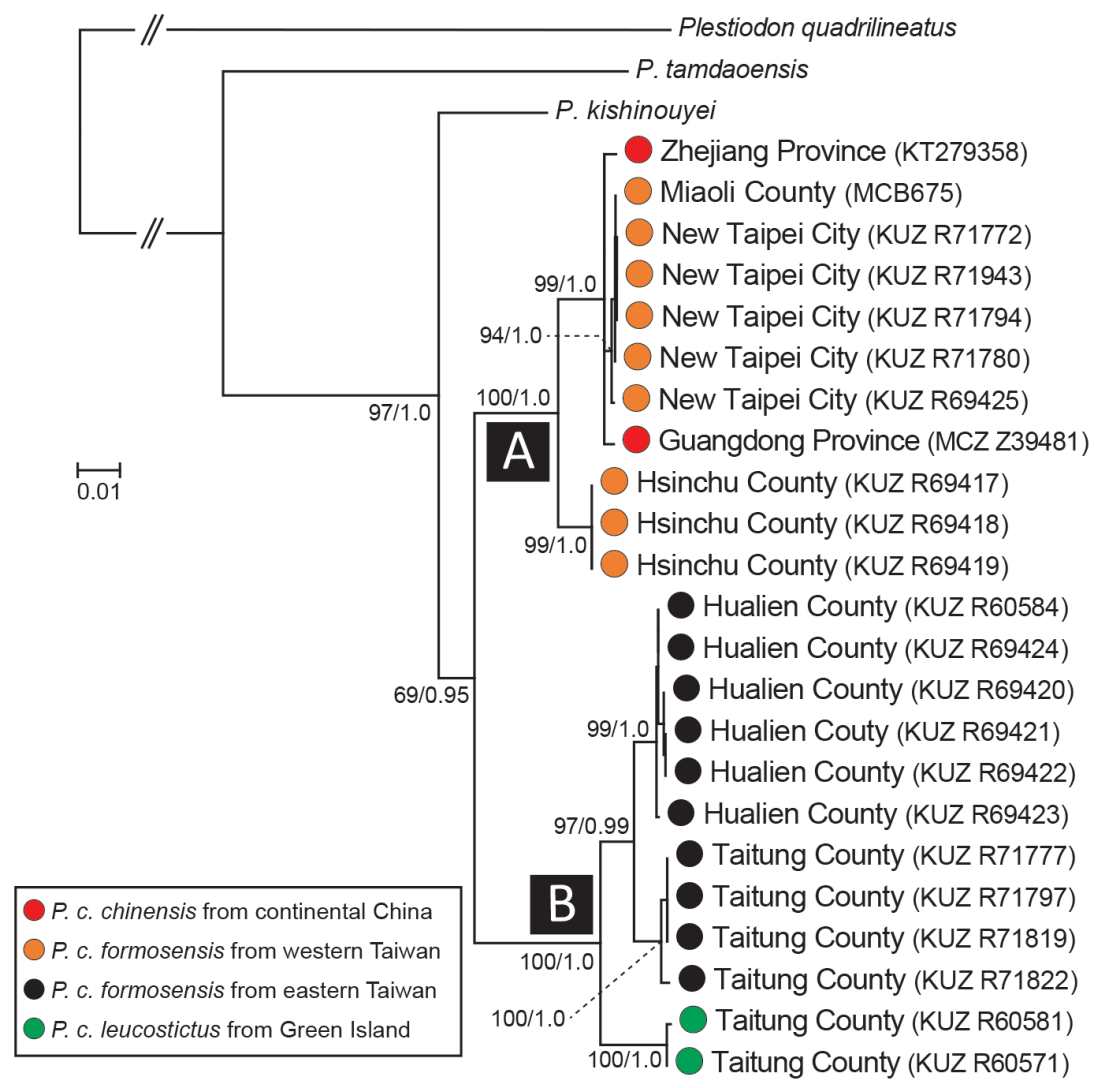
Maximum likelihood tree estimated using mitochondrial DNA sequences (2,351 bp). Numbers near interior branches show the bootstrap probabilities/Bayesian posterior probabilities. Sample names correspond to those given in Table 1.

The ML and BI trees were almost identical in topology. Therefore, we present only the ML tree in Fig. 3. The phylogeny did not support reciprocal monophyly between *P. c. formosensis* and *P. c. leucostictus*, but did demonstrate that *P. chinensis* from Taiwan is divided into two distinct clades (Clades A and B), although their sister relationship is not well supported in ML methods (69%/0.95 = ML bootstrap value/Bayesian posterior probability). Clade A contained *P. c. formosensis* from western Taiwan (noted as orange in Fig. 3), including the putative type locality (i.e. Xiangshan, Hsinchu County), together with *P. c. chinensis* from the southeastern region of continental China (red). Clade B contained *P. c. formosensis* from eastern Taiwan (black) and *P. c. leucostictus* from Green Island (green, topotypes). The mean uncorrected *p*-distance between Clades A and B was 5.1%/4.0% (= cyt *b*/ND1), which was comparable to the distances between these clades and their closest relative, *P. kishinouyei* (*i.e.* Clade A vs. *P. kishinouyei* = 5.7%/4.2%; Clade B vs. *P. kishinouyei* = 5.9%/4.6%).

**Figure 4.**
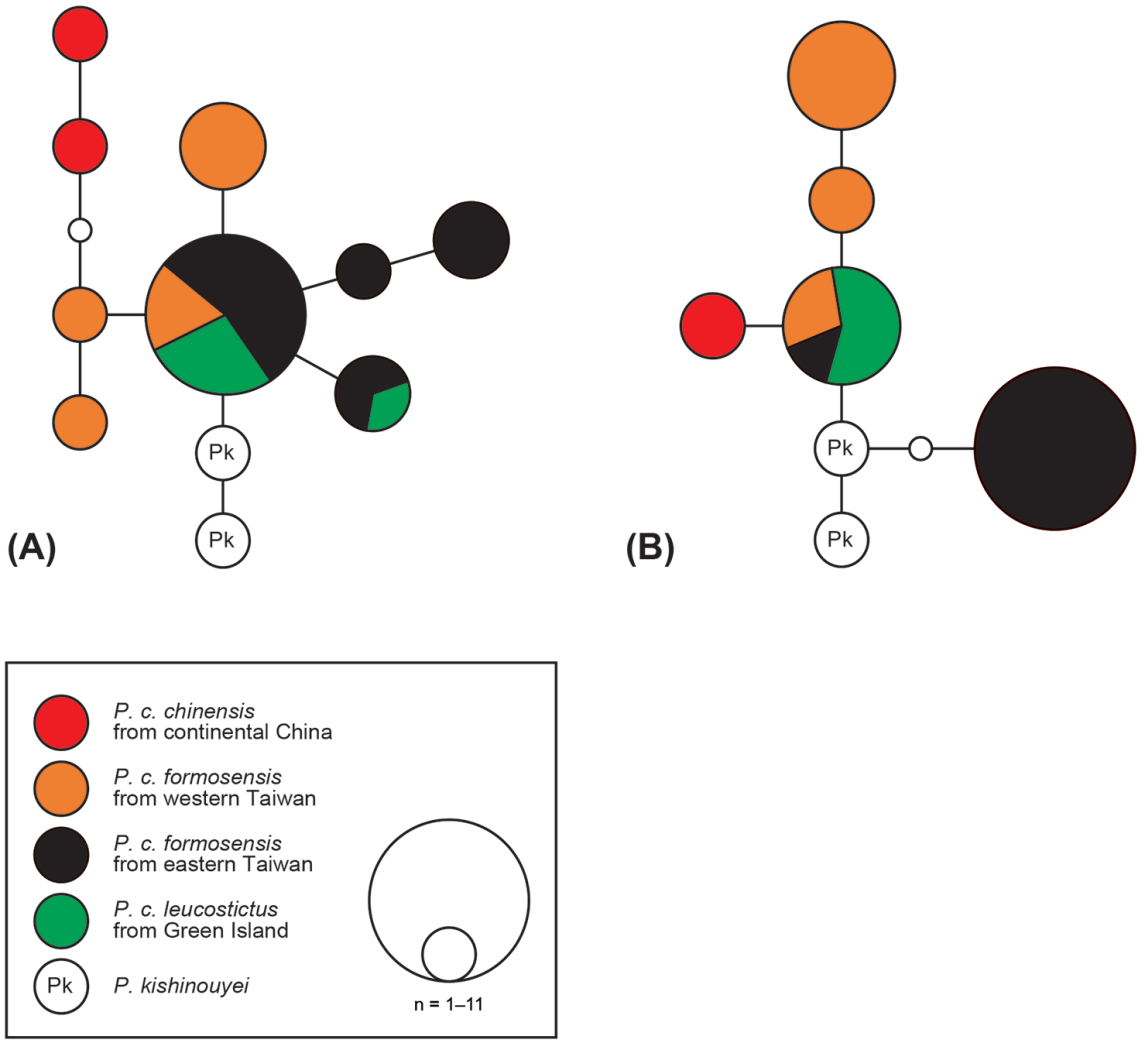
Median-joining networks of the RAG–1 **A** and PRLR **B** datasets. Open circles indicate missing alleles.

In nuclear DNA sequences, one individual from Jinshan (KUZ R71794) in the RAG–1 dataset was not phased with significant support (phase probability = 0.66), and was omitted. The remaining heterozygous sequences were inferred with phase probabilities ≥0.98. In the RAG–1 dataset, nine inferred alleles were obtained from *P. chinensis* (Fig. 4a). Of these, four alleles were obtained from eastern Taiwanese specimens. One of these four alleles was shared by specimens from western Taiwan, and two were shared by Green Island specimens. In the PRLR dataset, five inferred alleles were obtained from *P. chinensis* (Fig. 4b). Two alleles were found in specimens from eastern Taiwan, consisting of an allele endemic to this population and an allele shared with both specimens from western Taiwan and Green Island. The results of AMOVA for both nuclear datasets showed significant genetic differentiation between specimens from western Taiwan and eastern Taiwan plus Green Island (*F*_ST_ = 0.24, *P* = 0.0032 in the RAG–1 dataset; *F*_ST_ = 0.66, *P* < 0.0001 in the PRLR dataset). In contrast, there was no significant genetic differentiation between specimens from Taiwan (including western and eastern areas) and Green Island in either dataset (*F*_ST_ = −0.05, *P* = 0.5934 in the RAG–1 dataset; *F*_ST_ = 0.23, *P* = 0.0530 in the PRLR dataset).

## Discussion

The morphological investigation in this study confirmed that *P. chinensis* from eastern Taiwan are more similar in morphology to *P. c. leucostictus* than to *P. c. formosensis* from western Taiwan, especially in possessing a white spotted pattern without light lines on the dorsum (Fig. 2). Although the eastern Taiwanese specimens were similar to the western specimens in MSR, they showed a different frequency pattern from the western and Green Island specimens in PN (Table 2). Thus, the scale characters did not seem decisive as the basis of allocation of the eastern Taiwanese population. However, with the same coloration to *P. c. leucostictus* which is unique in this genus, the eastern Taiwanese populations should be assigned to this taxon. This morphologically based classification was also supported by molecular data: the genetic markers collectively suggested differentiation between western and eastern Taiwanese populations and close affinity between the eastern Taiwanese populations and *P. c. leucostictus* (Figs 3, 4). Therefore, the eastern Taiwanese and Green Island populations should be placed in a single taxon that is distinct from western Taiwanese populations. Based on the morphological differentiation of this taxon (Fig. 2) and a level of genetic differentiation comparable to that with another species (i.e. *P. kishinouyei*), we conclude that *P. c. leucostictus* should be recognized as a distinct species, i.e., *Plestiodon leucostictus* (Hikida, 1988).

According to the original description by Van Denburgh (1912a), the holotype of *P. c. formosensis* was collected from “San Shi Ka, Formosa”. The name probably derives from both Japanese and Taiwanese pronunciations. It signifies a “mountain foot” (Yi-Hung Chen, personal communication). Shang et al. (2009) proposed Xiangshan (Hsinchu County in western Taiwan) as the collection locality, while Zhao and Adler (1993) proposed Shanshang (Tainan County in western Taiwan) as the collection locality (sites 1 and 2 in Fig. 1, respectively). Based on our investigations on the island, this lizard is very abundant in northwestern, but very rare in southwestern Taiwan, indicating that the former place has a higher probability to be the real type locality. Unfortunately, we could not analyze specimens from both localities because population from the latter place was scarce. However, considering the continuous habitat and landscape pattern in western Taiwan, lizards from both localities should be assigned as the western taxon (Clade A). Furthermore, since the light lines was reported by Van Denburgh (1912b) from the holotype, it helps to confirm that *P. c. formosensis* should be applied only to the western lizard, while *P. leucostictus* stands for a valid specific name. Their disjunct distribution suggests that the Central Mountain Range in Taiwan may have played a major role as a natural barrier for these lowland lizards, congruent to the other phylogeographic cases which have shown huge diversification between eastern and western clades such as cobras (Lin et al. 2008), rhacophorid treefrogs (Lin et al. 2012), and lacertid lizards (Tseng et al. 2014, 2015).

Beyond the clear differentiation of *P. leucostictus* from other members of the *P. chinensis* complex, the intraspecific taxonomy of *P. chinensis* remains obscure. Subsequent to the description of *P. c. formosensis* by Van Denburgh (1912a), *P. chinensis* has been recognized as a polytypic species. Taylor (1936) placed *P. c. formosensis* in a junior synonym of the nominotypical subspecies without any comments, while he added a northern continental form, *P. c. pulcher*, to the subspecific rank of *P. chinensis*. He suggested that their geographic boundary might be located south of the Yangtze River. Hikida (1993) recognized the validity of *P. c. formosensis* and three other subspecies (*P. c. chinensis*, *P. c. leucostictus*, and *P. c. pulcher*), and regarded a Korean species, *P. coreensis* (Doi and Kamita 1937), as a synonym of *P. c. pulcher*. Zhao and Adler (1993) and Zhao et al. (1999) mostly agreed with Hikida’s (1993) classification, but did not recognize *P. c. pulcher* at subspecies rank. Instead, they regarded *P. c. daishanensis*, which was described by Mao (1983) based on specimens from the Zhoushan Islands, as a valid subspecies, although the other senior synonym, *P. c. rufoguttatus*, which was described by Cantor (1842) from another island in the same archipelago, is available for this taxon (Schmidt 1927, Taylor 1936).

As our study did not include representative specimens assigned to *P. c. pulcher*, *P. c. daishanensis* (or *P. c. rufoguttatus*), and *P. coreensis*, we could not undertake comprehensive revision of infraspecific taxonomy of *P. chinensis*. However, the low level of mtDNA genetic divergence we found between *P. c. formosensis* and *P. c. chinensis* (Fig. 3) supports their conspecific status, and *P. c. formosensis* should be assigned to *P. chinensis*. Additional detailed investigations of geographic variation in *P. chinensis*, including all of the putative nominal taxa, are needed to clarify the validity of the proposed subspecies in *P. chinensis*.

In this study, we solved the long and lasting debate to revise the taxonomic assignment of *Plestiodon* in eastern Taiwan from *P. c. formosensis* to *P. leucostictus*. The validity of *P. leucostictus* as a distinct species was confirmed, and we also revise its distribution from Green Island only to the eastern part of Taiwan. Nevertheless, this species in eastern Taiwan is far from common; Green Island is still the only locality for this species to show stable population size. In recent years, the dominant, large-sized skink *Eutropis multifasciata* was found to invade this island (Chen et al. 2009), which gave rise to a close tension from scientists. Although a project has been initiated to remove this invader, the problem requires sustained attention because of the notorious invasion history of this skink in Taiwan and other places (Ota et al. 1994, Witmer et al. 2007). On an islet like this size, invasive species, as well as other stochastic effects such as climate or demographic changes, may cause serious impacts to native species, and lead to negative consequence to this endangered taxon.

## Supplementary materials

Supplementary material 1 Appendices Data type: Adobe Acrobat (pdf) Explanation note:
**Appendix I**: Specimens examined in the morphological analysis of this study
**Appendix II**: Primers used in the PCRs of this study.
**Appendix III**: MtDNA sequence partitions and the best-fit models for phylogenetic analysis. This dataset is made available under the Open Database License (http://opendatacommons.org/licenses/odbl/1.0/). The Open Database License (ODbL) is a license agreement intended to allow users to freely share, modify, and use this Dataset while maintaining this same freedom for others, provided that the original source and author(s) are credited.Kazuki Kurita, Yukiko Nakamura, Taku Okamoto, Si-Min Lin, Tsutomu Hikida
