# Supplementary material for: Taxonomic reassessment of two subspecies of Chinese skink in Taiwan based on morphological and molecular investigations (Squamata, Scincidae)
Source: Zookeys. 2017 Aug 2;(687):131–48. doi: 10.3897/zookeys.687.12742 (PMC5672577; doi:10.3897/zookeys.687.12742)
Supplement: Supplementary material 1 — Appendices [file zookeys-687-131-s001.pdf]

Appendix I

Specimens examined in the morphological analysis of this study (all from Taiwan). Museum abbreviations are as follow: CAS (California Academy of Sciences) and KUZ (Zoological Collection of the Kyoto University Museum).

Plestiodon chinensis formosensis

"San Shi Ka": CAS 18605 (holotype), CAS 18604, 18606 (paratypes); New Taipei City: Jinshan: KUZ R71771–75, 71780, 71792–94, 71817, 71940–41, 71943, 71987, 72027; New Taipei City: Bali: KUZ R69425; New Taipei City: Sanchong: KUZ R51452; Keelung City: Keelung: CAS 18603 (paratype); Keelung City: Keelung Island, KUZ R60638–39; Taipei City: Waishuanghsi: KUZ R51443–44, 51449–51, 51453; Hsinchu County: Hsinchu City: KUZ R45087; Hsinchu County: Xiangshan: KUZ R69417–18; Miaoli County: Zhunan,: KUZ R21313, 70946, 70948–53, 70955–56, 70959; Hualien County: Hualien City: KUZ R69420–21; Hualien County: Guangfu: KUZ R69423–24; Hualien County: Ruisui: KUZ R51447; Taitung County: Sansiantai: KUZ R71645, 71776–78, 71795–98, 71818–23, 71942, 71991–71992, 72024–26; Taitung County: Luye: KUZ R60637.

Plestiodon c. leucostictus

Taitung County: Green Island: KUZ R7239, 7293–94, 7300–02, 7310, 7333, 7356, 8444, 35029, 35039, 35044, 53120, 53199, 60450–51, 60542, 60571–76, 60578, 60582, 60584.

Appendix II

Primers used in the PCRs of this study. See text for gene composition in each fragment.

Gene	Primer name	Sequence (5′-3′)	Reference
Cyt *b* and its adjacent	cytb-outF	CCACCGTTGTTTTCAACTACA	Kurita and Toda (2017)
Cyt *b* and its adjacent	H15752	TACTGGTTGACCACCGATTCAAGT	Richman and Price (1992)
Cyt *b* and its adjacent	L15192cbEu[†]	TGAGGCGCAACCGTAATTACAAACCT	Okamoto and Hikida (2009)
Cyt *b* and its adjacent	H15263cbEuki[†]	TGGAATGTGAAAAATCGGGTGAGRGTWGC	Kurita and Toda (2017)
ND1 and its adjacent	16dR	CTACGTGATCTGAGTTCAGACCGGAG	Leaché and Reeder (2002)
ND1 and its adjacent	tMet	TCGGGGTATGGGCCCRARAGCTT	Brandley et al. (2011)
ND1 and its adjacent	ND1-INTFm1[†]	ACAYTRGCYGARACYAAYCGAGCACC	Kurita and Toda (2017)
ND1 and its adjacent	ND1-INTRm1[†]	TAYACDGCYATGCTTGARAGGGCTA	Kurita and Toda (2017)
RAG1	RAG1SK-F1362	CTTGGCAATCCGAGTCAACACCTTTCTCAG	Brandley et al. (2011)
RAG1	H-RAG1b	GACTGCCTGGCATTCATTTT	Kearney and Stuart (2004)
RAG1	L-mRAG1Pl[†]	AACTGTTCCTTCAATAGATGC	Kurita and Hikida (2014b)
RAG1	RAG1SK-R2054[†]	GCCCTCTACTTCACGGACAAGCTTTTCATC	Brandley et al. (2011)
PRLR	PRLR_f1	GACARYGARGACCAGCAACTRATGCC	Townsend et al. (2008)
PRLR	PRLR_r3	GACYTTGTGRACTTCYACRTAATCCAT	Townsend et al. (2008)

[†]Internal primers used only for sequence reaction.

Appendix III

MtDNA sequence partitions and the best-fit models for phylogenetic analysis.

Partition	*Plestiodon chinensis* and outgroup taxa		
	AIC model for ML analysis	AIC model for BI analysis	Number of characters
Cyt *b* 1st codon position	HKY85 + G	K80+G	336
Cyt *b* 2nd codon position	HKY85 + G	HKY85+G	336
Cyt *b* 3rd codon position	J3 + G	GTR+G	336
ND1 1st codon position	TN93+G	K80+G	322
ND1 2nd codon position	HKY85 + I	HKY85+I	322
ND1 3rd codon position	TN93 + I	GTR+I	322
16S rRNA	HKY85	K80	146
All tRNAs (Leu, Ile, Gln, Met)	HKY85 + G	HKY85+G	231
